# Ultrasound at the patient’s bedside for the diagnosis and prognostication of a renal colic

**DOI:** 10.1186/s13089-021-00246-2

**Published:** 2021-11-22

**Authors:** Jean-Eudes Bourcier, Emeric Gallard, Jean-Philippe Redonnet, Morgan Abillard, Quentin Billaut, Laura Fauque, Anna Jouanolou, Didier Garnier

**Affiliations:** 1 Emergency, Anesthesiology and Critical Care Department, Lourdes Hospital, Lourdes, France; 2 Pôle Anesthésie-Réanimation-Urgences, Hôpital de Lourdes, 2 Avenue Alexandre Marqui, 65100 Lourdes, France

## Abstract

**Background:**

Diagnosing a ureteral colic is sometimes difficult; however, clinicians should not fail to detect a surgical emergency. This is why diagnostic strategies depend on the imaging examinations, especially ultrasound. Prior studies have investigated the accuracy of Point of Care Ultrasound (PoCUS), but there are relatively few. This study aimed to evaluate the performance of the PoCUS in the diagnosis of renal colic. The secondary objective was to evaluate the relationship between the imaging results and the treatment performed.

**Methods:**

After the clinical evaluation of patients aged > 18 years with suspected ureteral colic, the Emergency Physician (EP) trained in ultrasound performed PoCUS to conclude whether a diagnosis of “renal colic” should be made. A computed tomography (CT) examination was subsequently performed, to determine whether ureteral or bladder lithiasis was present to diagnose a ureteral colic. The patient’s management was decided according to the to degree of urinary tract dilatation, presence of perinephric fluid, size, and localization of stones.

**Results:**

Of the 12 Eps in our units, seven met the training criteria for the inclusion of patients. A total of 103 patients were analyzed, and the renal colic diagnosis was retained in 85 cases after the CT examination. The accuracy of PoCUS was 91% (86; 95%) for detecting urinary tract dilatation, 83% (76; 90%) for detecting perinephric fluid, and 54% (44; 64%) for detecting lithiasis. Only high urinary tract stones with ≥ 6 mm diameter were surgically managed (*p* < 0.01). Conversely, distal ureteral stones with a diameter of < 6 mm were managed with medical ambulatory treatment (*p* < 0.05).

**Conclusion:**

PoCUS is a good diagnostic tool, for renal colic, and could help reduce the requirement for the CT examinations and, hence, reduce induced radiation exposure.

## Introduction

Ureteral colic, which is usually revealed by the occurrence of acute lumbar or abdominal pain accounts for 1–5% of the admissions in emergency Unit [1]. Some of its clinical symptoms are similar to those of other pathologies, such as appendicitis, renal infarction, or aortic aneurysm fissuration. Furthermore, in 5% of the cases, ureteral colic is complicated by condition, such as obstructive acute renal failure, suppurated urinary retention, and rupture of the urinary tract, and may require emergency drainage [2, 3]. This is why imaging is often necessary from the initial stage of the patient’s management. Computed tomography (CT) without the injection of a contrast agent is the gold standard imaging modality; however, its reasonable use is necessary owing to the involved radiation exposure [4]. Moreover, CT equipment or a radiologist is not always immediately available [5]. Point of Care Ultrasound (PoCUS), performed by the emergency physician (EP), eliminates these drawbacks; however, its reliability remains to be demonstrated [6, 7].

In addition, although imaging is recommended, the impact on patient management is unclear [8–10].

Therefore, the aim of this study was to compare the performance of PoCUS performed by the emergency physician relative to a CT scan interpreted by a radiologist.

The secondary goal was to evaluate the impact of the imaging findings on the patients management.

## Patients and methods

### Study design

This was a 1-year, single-center, prospective observational study in an emergency unit recording 19,000 visits per year.

### Ethic approval statement

The study protocol was approved by ethics Committee of our institution (PV 170216), according to the Jardé law (France). The IRB (Institutional Review Board) considered that the standard of care was not modified. Indeed, in our institution, we did not have access to ultrasound performed by a radiologist. That is why CT scan was the first line examination in case of suspected nephritic colic.

### Patients

Any patient aged ≥ 18 years old presenting at the emergency department with a nontraumatic pain suggesting a ureteral colic (i.e., lumbar and/or pelvic pain that suddenly appeared, with or without pollakiuria and hematuria) was considered eligible, by EP. Patients were included if their attending physician was trained in clinical ultrasound.

Physician qualification was based on the completing a 5-day theoretical and practical training session at a certified center, followed by 18 months of e-learning. This course was in accordance with the recommendations of the American College of Emergency Physicians [11].

Pregnant women and patients with the previous imaging examinations were excluded. Patients who did not have an imaging session or without imaging report were also excluded.

### Clinical–biological data

After the patient interview and clinical examination, the patients received an analgesia and underwent routine blood and urinary examinations.

### Ultrasound data

The EP in charge of the patient performed the ultrasound examination using an Xporte© SonoSite device (SonoSite, Bothell, WA, USA). A convex abdominal probe (3.5–5 MHz) was used, according to a longitudinal grid technique (with the probe parallel to the plane of the bed). The EP followed the two axillary lines, to analyze the epigastric region, and the under-umbilical area. This permitted analyzing both the kidneys and ureters using low longitudinal and transverse intercostal slices, to measure and compare the pyelic and caliceal cavities.

The EP searched for dilatation of the pyelocalyceal cavities,which was graded according to severity, as follow: grade 1, pyelic dilatation alone; grade 2 with confluent calyceal dilatation of > 1 cm; grade 3 same as grade 2, but with a confluent dilatation of 1.5 cm diameter; grade 4 same as grade 3, but with additional cortical thinning. Grade 3 or 4 dilatation was considered severe [12]. An equal focus was given to detect peri-renal effusion, which indicates a rupture of the excretory tract.

**Fig. 1:**
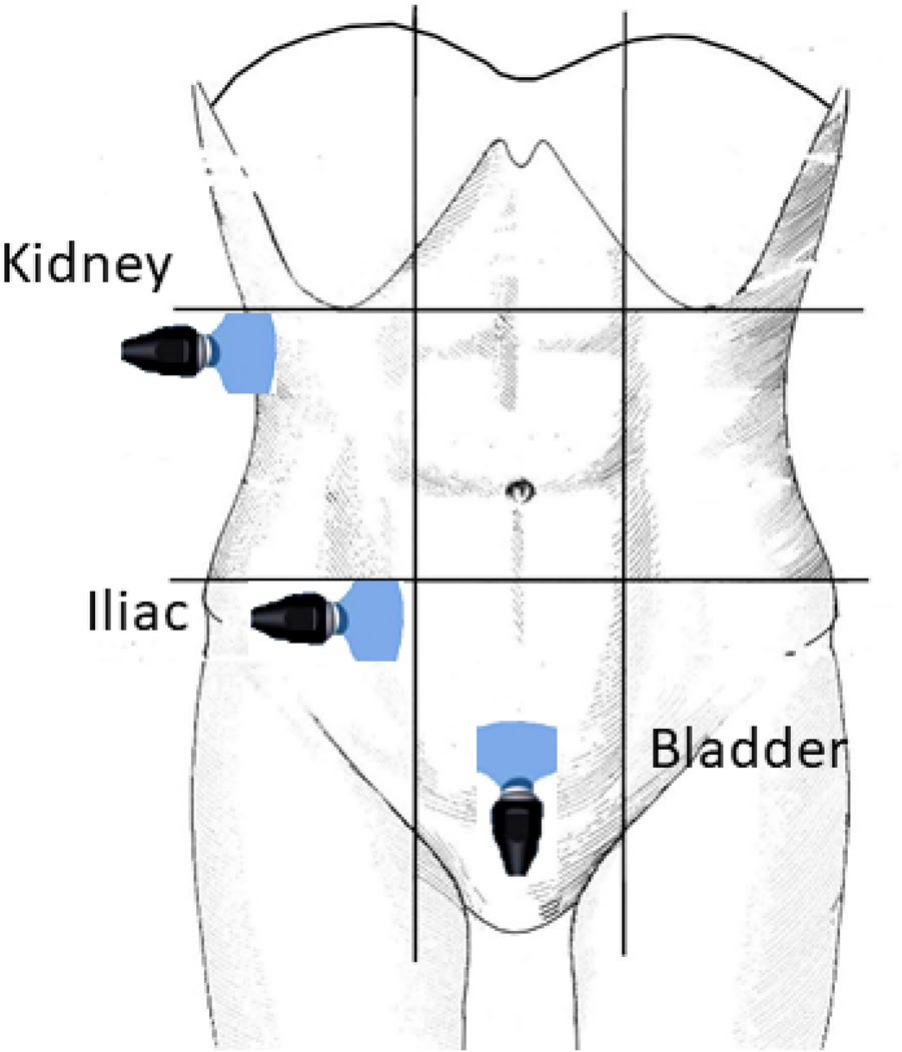
Successive probe positions to explore urinary tract

**Fig. 2:**
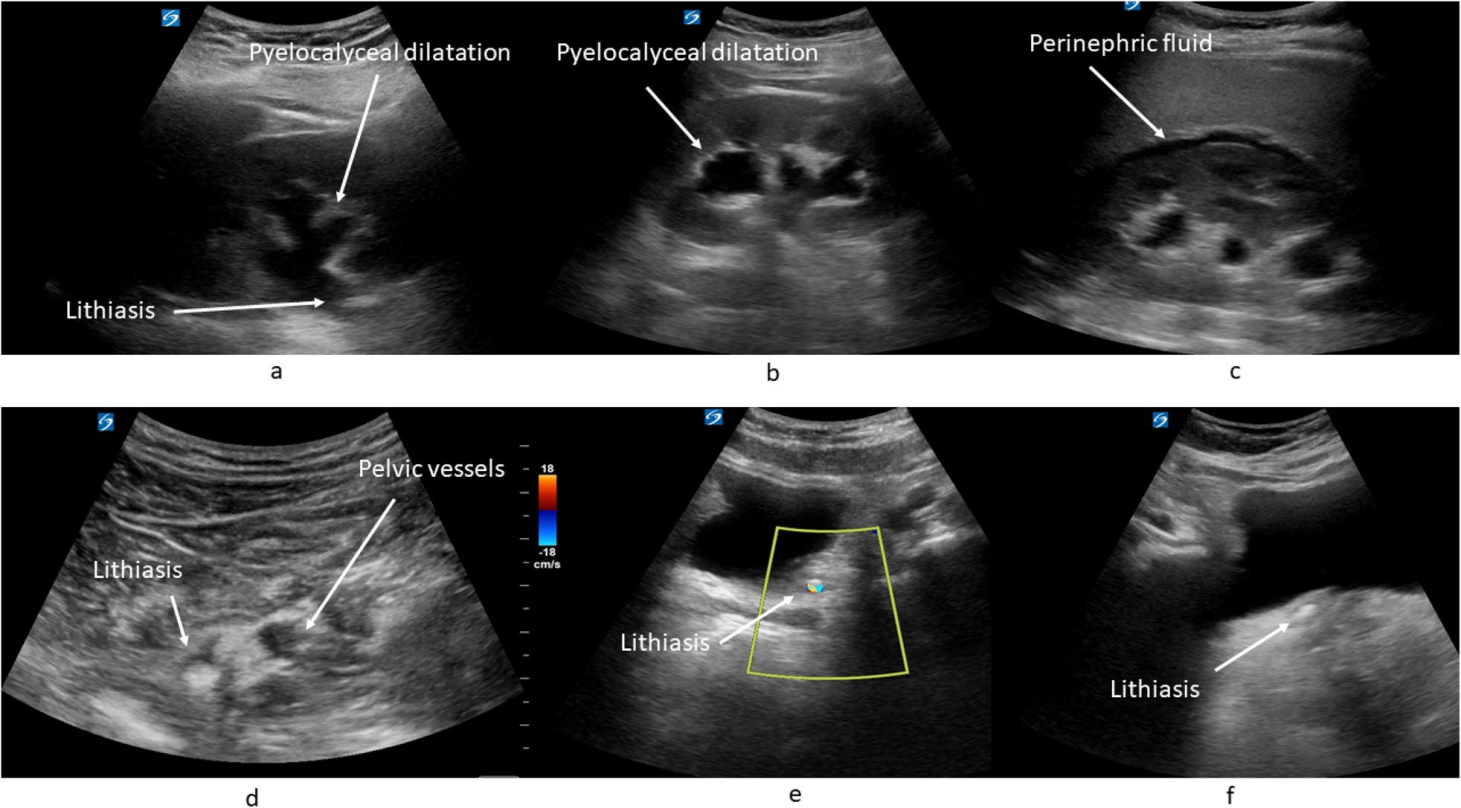
Ultrasound images: **a** pyelocalyceal dilatation and proximal lithiasis, **b** pyelocalyceal dilatation, **c** perinephric fluid, **d** pelvic lithiasis, **e** bladder lithiasis and twinkle artifact in color doppler; and **f** bladder lithiasis

The proximal ureter was evaluated to detect lithiasis in the pyelo-ureteral junction. The EP also ultrasonologically examine the area next to the iliac vessels and the supra pubic region, to detect lithiasis in the iliac or pelvic ureter, or in uretero-vesical meatus (Figs. 1 and 2).

The ultrasound examination also included the detection of peritoneal effusion and measurement of the abdominal aortic caliber to exclude an aneurysm.

Once PoCUS was completed, the EP wrote a report of the examination findings, including: the degree of dilatation, presence, or absence of lithiasis including the size and location of stone, if present; and the presence or absence of perinephric fluid. Finally, the EP concluded whether the patient had “ureteral colic or not”.

### CT scan data

After PoCUS performed, all patients underwent an abdominopelvic CT without contrast examination with TOSHIBA© Aquilion Prime (Canon Medical System Coroporation, Otowara, Japan), the induced dose was 213 milligray. A radiologist who was blinded to the ultrasound examination result checked for the presence or absence of pyelocaliceal dilatation, perirenal effusion, or ureteral lithiasis, reporting the location and size of the stones, if present. Finally, the radiologist concluded whether or not “the patient had renal colic”.

### Ureteral colic diagnosis

The diagnosis of ureteral colic was retained when ureteral lithiasis with or without an upstream dilatation or bladder lithiasis was detected on CT. It was also retained if the expulsion of the stones had been clinically confirmed.

### Judgment criteria

The primary end point of the study was the diagnostic agreement rate between PoCUS and CT.

The secondary objective was to evaluate the relationship between the result of the ultrasound examination and the treatment performed.

### Statistical analysis

A physician not involved in the study, but collected the results of the examinations (PoCUS and CT scan) for analysis and comparison.

The data were analyzed using Excel © software. Quantitative variables are expressed as mean ± standard deviation. Qualitative variables are expressed as number and percentage. The performance of PoCUS in detecting of pyelocalyceal dilatation, perirenal fluid, and ureteral lithiasis was expressed as sensitivity, specificity, positive predictive value, negative predictive value, and accuracy. Accuracy was defined according to the proportion of confirmed cases, or the ratio of true positives and true negatives to the total population. Further 95% confidence intervals (95% CI) were calculated.

To assess the impact of imaging on treatment, a Fischer’s exact test was performed for severe dilatation of the urinary tract, perinephric fluid, and size and location of stones, with a significance level set at 0.05.

## Results

### Patient characteristics

Of the 12 EPs on duty, seven met the training criteria for the inclusion of patients.

**Fig. 3:**
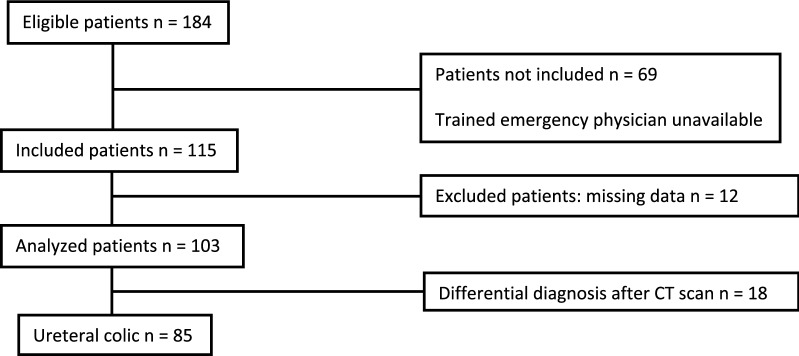
Patient flowchart

A total of 184 patients suspected to have renal colic from July 2017 to June 2018 were eligible, and 103 patients were finally analyzed (Fig. 3). The patients’ demographic data are reported in Table 1.

**Table 1:** Characteristics of patients with confirmed ureteral colic

	Ureteral colic (*n* = 85)	Differential diagnosis (*n* = 18)
Age years (mean ± standard deviation)	52.1 ± 15.9	52 ± 12.3
Sex		
Female	28 (33%)	11 (61%)
Male	57 (67%)	7 (39%)
Antecedents		
Ureteral colic	38 (44%)	6 (33%)
Uropathy	6 (7%)	4 (22%)
Pain relief		
Level 1	44 (52%)	8 (44%)
Level 2	11 (13%)	5 (28%)
Level 3	28 (34%)	5 (28%)
NA	2 (2%)	0
Pain duration before admission		
< 6 h	51 (60%)	4 (22%)
6–24 h	6 (7%)	5 (28%)
> 24 h	20 (24%)	9 (50%)
NA	8 (9%)	0
Size of stone, mm		
< 6	60 (70%)	0
> 6	25 (30%)	0
Location of stone		
Pyelo-ureteral junction	7 (8%)	0
Lumbar ureter	21 (25%)	0
Lower third of the urinary tract	57 (67%)	0
Hospitalization	16 (18%)	2 (11%)

NA, not applicable

### Main results

Clinical and ultrasound examinations revealed that 18 patients were mistakenly suspected of having ureteral colic. Further, in 15 of these 18 patients, ultrasound examinations mainly found pyelocalyceal dilatation. The diagnoses after the CT were as follow: pyelonephritis (*n* = 4), kidney neoplasia (*n* = 1), adrenal hematoma (*n* = 1), renal cyst (*n* = 1), prostatism (*n* = 1), uncomplicated diverticulitis (*n* = 2), uncomplicated appendicitis (*n* = 1), nonspecific inflammation of the digestive tract (*n* = 2), uncomplicated ovarian cyst (*n* = 1), nonspecific abdominal pain (*n* = 3), and low back pain (*n* = 1). PoCUS detected one case of ovarian cyst and one case of diverticulitis. Before CT, ultrasound examinations did not exclude any diagnosis of ureteral colic. The PoCUS finding compared to CT are reproted in Table 2.

**Table 2:** PoCUS findings compared to CT

	CT		
	Pyelo-caliceal dilatation +	Pyelo-caliceal dilatation −	Total
PoCUS			
Pyelo-caliceal dilatation +	83	5	88
Pyelo-caliceal dilatation −	4	11	15
Total	87	16	103

	CT		
	Perinephric fluid +	Perinpehric fluid −	Total
PoCUS			
Perinephric fluid +	12	8	20
Perinephric fluid −	9	74	83
Total	21	82	103

	CT		
	Stone +	Stone −	Total
PoCUS			
Stone +	34	2	36
Stone −	45	22	67
Total	79	24	103

Eight patients diagnosed with renal colic had no ureteral lithiasis on CT, but had pyelocalyceal cavity dilatation. Six of them had bladder lithiasis and two had spontaneous elimination of stones between the PoCUS and CT examinations.

The performance of PoCUS in detecting the abnormalities sought as compared to CT is reported in Table 3.

**Table 3:** Summary of diagnosis performance of PoCUS

	Pyelocalyceal dilatation	Lithiasis	Perinephric fluid
Se [95% CI]	95 [89–100]	43 [32–54]	57 [36–78]
Sp [95% CI]	68 [58–77]	92 [80–100]	90 [83–97]
PPV [95% CI]	94 [88–99]	94 [87–99]	60 [38–81]
NPV [95% CI]	73 [63–82]	33 [21–44]	89 [82–95]
A [95% CI]	91 [86–95]	54 [44–64]	83 [76–90]

Se, sensitivity; Sp, specificity; PPV, positive predictive value; NPV, negative predictive value; A, accuracy

Among the 85 patients diagnosed with ureteral colic, 43 had lithiasis not detected on ultrasound (14 in the bladder–ureteral junction, six in the pelvic ureter, 21 in the lumbar ureter, and two in the proximal ureter) (Table 4).

**Table 4:** Patients’ management and imaging findings on CT

Pathological imaging findings	Surgical management < 24 h (*n* = 16)	Ambulatory management (*n* = 69)	Total (*n* = 85)	*p*
Perinephric fluid	9	12	21 (24%)	< 0.05
Severe pyelocalyceal dilatation	6	9	15 (17%)	< 0.05
Location and size of stones				
Proximal > 6 mm	12^a^	4	16 (19%)	< 0.05
Proximal < 6 mm	2	10	12 (15%)	1
Distal > 6 mm	2	7	9 (10%)	0.67
Distal < 6 mm	0	48	48 (56%)	< 0.05

^a^Three patients who initially received ambulatory treatment were readmitted within 48 h and underwent surgical treatment

Sixteen patients underwent surgical treatment in the emergency setting, three of whom had ureteral obstruction and urinary sepsis (Table 3).

Among the nine patients who presented with pelvic stones > 6 mm, two underwent urgent drainage. Among the seven remaining patients, four were secondarily treated with extracorporeal lithotripsy.

Among the 69 outpatients, 3 (4%) had a secondary readmission for a recurrent pain and four others underwent delayed lithotripsy.

## Discussion

This study assesses the performance of PoCUS compared with CT in the simultaneous detection of urinary tract dilatation, lithiasis, and perirenal effusion. Our results suggest that ultrasound allows the reasonable use of CT a secondary intervention when the ultrasound findings are inconclusive.

In this work, the accuracy of PoCUS in detecting a pyelocalyceal dilatation was 91%. This is comparable to what is reported in the literature, regardless of whether the examination was performed by an EP or a radiologist [13–15]. Overestimations occur owing to the difficulty posed by structure, such as Malphigi pyramids, para-pyelic cysts, or cortical cysts, which can be incorrectly interpreted as a dilatation of the urinary tract (not central confluents findings). Moreover, ultrasound examinations were performed ≤ 6 h after the beginning of symptoms in 50% of the patients, explaining the false negative results due to delayed dilatation. Meanwhile, the dilatation found on ultrasound was sometimes due to other diseases such as appendicitis, colic inflammation, and kidney or pelvic mass. However, PoCUS did not exclude the diagnosis of renal colic. It would require widening the exploration of the digestive tract, with a high-frequency probe.

The accuracy of BUS in detecting a perinephric fluid was 83%, and we found no data in the literature about this topic.

The performance of PoCUS in detecting lithiasis, and therefore ureteral colic was as follow: accuracy, 54%; sensitivity, 43%; and specificity, 92%. These results are comparable to available data from examination performed by a radiologist [16–18]. Our study is the only study to involve an EP in this setting. Among the 43 lithiasis cases not detected by ultrasound, 14 were located in the pelvic ureter or at the junction between bladder, and ureter and their detection could have been improved. An explanation for the failed detection was that bladder was often empty at the time of PoCUS owing to prior completion of a urine dipstick test.

In our study, a urinary bypass was performed only for stones ≥ 6 mm in diameter located in the proximal or lumbar ureter. Patients with stones < 6 mm were treated as outpatients.

Severe dilatation of the urinary tract (grade 3 or 4) was correlated with hospital stay in many studies [19], as in ours. However, in our study, there was a relationship between perinephric fluid and urgent surgical treatment. In the literature, this point is not clarified [10, 20].

In summary, our results suggests that findings on the ultrasound correlate well with the CT and ultimate surgical treatment such that the ED physician may be able to make an early decision regarding the referral pathway and possible discharge of patients with small distal stones [21, 22]. PoCUS could be sufficient to manage a subset of uncomplicated patients unlikely to require further surgical management. A “clinical-ultrasound” step can be recommended, as 40% of lithiasis cases were detected by PoCUS in our study. CT could be performed only as a secondary intervention in the other patients, to avoid radiation exposure and reduce the cost in this patient population [23, 24].

Moreover, PoCUS is immediately available after admission, thus allowing early evaluation, and is more comfortable and safer than CT. In addition, it does not require patients to be transferred from the emergency unit, which is beneficial for more severe cases.

Our study had some limitations. This was a single-center study involving a relatively small number of patients, in which 37% of eligible patients were not included because their EP did not meet the training criteria. Further, it would have been interesting to evaluate the patients’ length of stay in the emergency unit, as some studies demonstrated a clear decrease in this setting [25].

**Fig. 4:**
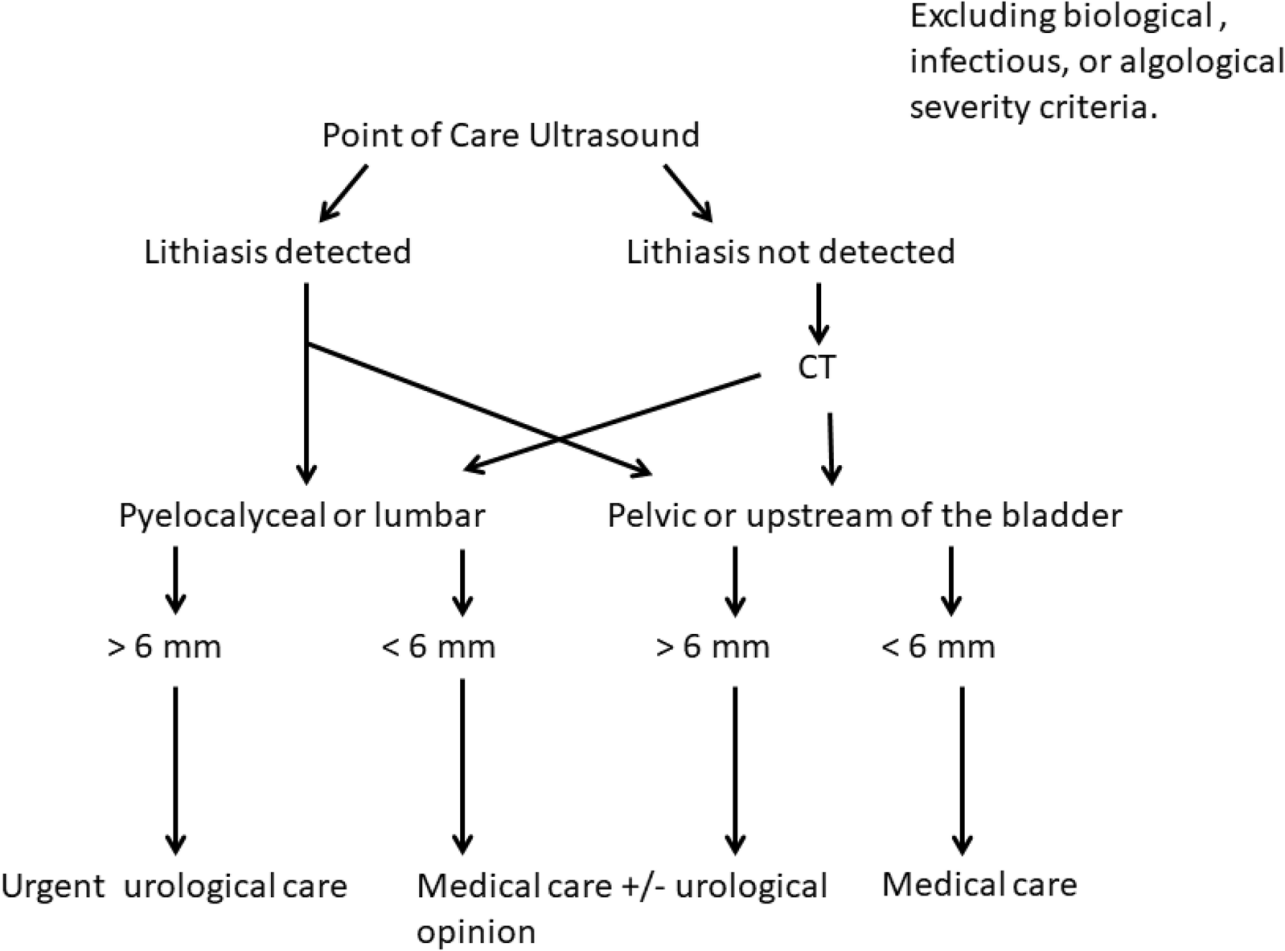
Use of imaging (PoCUS and CT) for the management of patients presenting with ureteral colic

Our results suggest that patient management could follow the algorithm shown in Fig. 4 involving the complementary use of BUS and CT for detecting lithiasis.

## Conclusion

PoCUS is a good tool for the diagnostic and therapeutic evaluation of patients for renal colic. Its availability at the bedside, in addition to allowing the reasonable use of CT could optimize patient care, particularly in the most acute cases.

## Data Availability

No additional data available.

## Abbreviations

EP: Emergency physician
BUS: Bedside ultrasound
CT: Computerized tomography
PPV: Positive predictive value
NPV: Negative predictive value
Se: Sensitivity
Sp: Specificity
